# Ontogeny of adrenal-like glucocorticoid synthesis pathway and of 20α-hydroxysteroid dehydrogenase in the mouse lung

**DOI:** 10.1186/1756-0500-7-119

**Published:** 2014-03-01

**Authors:** Eric Boucher, Pierre R Provost, Yves Tremblay

**Affiliations:** 1 Reproduction, mother and youth health, Centre de recherche du CHU de Québec, Québec, QC, Canada; 2 Department of Obstetrics/Gynecology & Reproduction, Faculty of Medicine, Université Laval, Québec, QC, Canada; 3 Centre de Recherche en Biologie de la Reproduction (CRBR), Faculté de Médecine, Université Laval, Québec, QC, Canada

**Keywords:** 21-hydroxylase, 11β-hydroxylase, Corticosterone, Development, Fetal, Glucocorticoid, Progesterone, Steroidogenesis, Mineralocorticoid, 11β-HSD

## Abstract

**Background:**

Glucocorticoids exert recognized positive effects on lung development. The genes involved in the classical pathway of glucocorticoid synthesis normally occurring in adrenals were found to be expressed on gestation day (GD) 15.5 in the developing mouse lung. Recently, expression of two of these genes was also detected on GD 17.5 suggesting a more complex temporal regulation than previously expected. Here, we deepen the knowledge on expression of “adrenal” glucocorticoid synthesis genes in the mouse lung during the perinatal period and we also study expression of the gene encoding for the steroid inactivating enzyme 20α-hydroxysteroid dehydrogenase (20α-HSD).

**Results:**

We performed an ontogenic study of P450scc, 3β-hydroxysteroid dehydrogenase/Δ^5^-Δ^4^ isomerase 1 (3β-HSD1), 21-hydroxylase, 11β-hydroxylase, 11β-HSD1, and 11β-HSD2 expression up to post natal day (PN) 15. The substrate (progesterone) and the product (deoxycorticosterone) of 21-hydroxylase are substrates of 20α-HSD, thus 20α-HSD (*Akr1c18*) gene expression was investigated. In lung samples collected between GD 15.5 and PN 15, 11β-hydroxylase was only detected on GD 15.5. In contrast, all the other tested genes were expressed throughout the analyzed period with different temporal expression patterns. P450scc, 21-hydroxylase, 20α-HSD and 11β-HSD2 mRNA levels increased after birth with different patterns including an increase from PN 3 with a possible sex difference for 21-hydroxylase mRNA. Also, the 21-hydroxylase protein was observed by Western blot in perinatal lungs with higher levels after birth.

**Conclusion:**

Progesterone is present at high levels during gestation and the product of 21-hydroxylase, deoxycorticosterone, can bind the glucocorticoid receptor with an affinity close to that of corticosterone. Detection of 21-hydroxylase at the protein level during antenatal lung development is the first evidence that the adrenal-like glucocorticoid synthesis pathway detected during lung development has the machinery to produce glucocorticoids in the fetal lung. Glucocorticoids from lung 21-hydroxylase appear to modulate lung ontogenesis through paracrine/intracrine actions.

## Background

Glucocorticoids (GCs) play an essential role in fetal lung development [1,2]. They exert various effects including thinning of alveolar septa, increase in the number of type I pneumonocytes, decrease in pneumonocyte cell division, and stimulation of secretion of fibroblast paracrine factors that play a role in type II pneumonocyte maturation [3]. Glucocorticoid receptor-deficient mice were reported to die within a few hours after birth because of respiratory failure [4], showing the importance of GCs in the lung development process. The requirement for GCs in normal lung development was also demonstrated with a CRH knockout (KO) mouse model. CRH KO homozygous fetuses from CRH KO homozygous mothers were delivered normally at term, but died on the first day of life with an overall failure in lung development, except when GCs were administered to pregnant dams [2,5]. The overall failure of lung development was characterized by hypercellularity of the mesenchymal compartment and decreased expression of mRNAs associated with mature lung epithelial cells such as those of surfactant protein (SP)-A, SP-B and CCP10.

**Figure 1:**
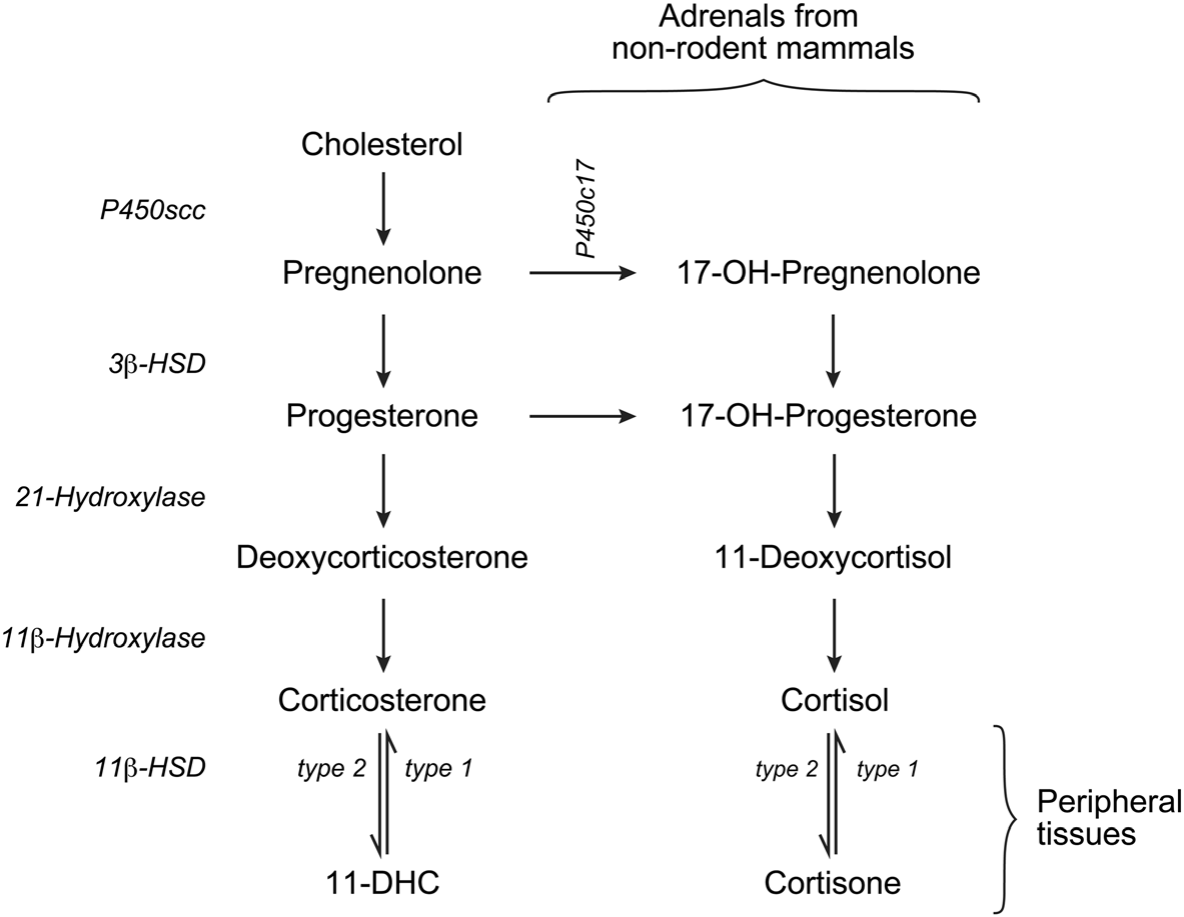
**Glucocorticoid synthesis pathway in rodent and non-rodent mammals.** Corticosterone (rodent) and cortisol (non-rodent mammals) are the most potent circulating GCs. Their production from cholesterol in adrenals correspond to the classical pathway of GC synthesis. There is no P450c17 enzyme in the rodent adrenal. Cell-specific 11β-HSD-catalyzed reactions occur in peripheral tissues. During the last decade, expression of some or all the enzymes catalyzing GC production from cholesterol has been observed in some non-adrenal tissues including the developing lung.

The endogenous GCs accessible to the developing lung originate from two sources. They can be provided by adrenals through the classical pathway of GC synthesis from cholesterol, or by the lung where cortisone or 11-dehydrocorticosterone (11-DHC, rodent) can be reactivated to cortisol or corticosterone, respectively. In the rodent adrenals, cytochrome P450 side-chain cleavage (P450scc), 3β-hydroxysteroid dehydrogenase/Δ^5^-Δ^4^ isomerase (3β-HSD), 21-hydroxylase, and 11β-hydroxylase are involved in GC synthesis (Figure 1). All these enzymes play a similar role in the adrenals of the human and many other non-rodent mammals, except that cytochrome P450c17 converts pregnenolone and progesterone into 17OH-pregnenolone and 17OH-progesterone, respectively. This leads to the production of the active GC cortisol instead of corticosterone. In peripheral tissues including the lung, GC synthesis was considered for many years to be limited to cortisol (human) and corticosterone (rodents) re-activation by 11β-hydroxysteroid dehydrogenase type 1 (11β-HSD1) [6] following their prior inactivation by 11β-HSD2. This perception is changing since genes normally involved in the classical adrenal pathway of GC synthesis were found to be expressed in some peripheral sites such as thymus, skin, and brain [7]. We are among the pioneers that have described expression of the adrenal genes outside adrenals [8].

In 2005, we reported that the four genes encoding for the enzymes catalyzing corticosterone synthesis from cholesterol and StAR were expressed in the fetal lung on gestation day (GD) 15.5 in the Balb/c mouse [8]. StAR is essential to this synthesis pathway since it plays a role in the intramitochondrial movement of cholesterol [9]. In fetal lungs studied up to GD 18.5, 11β-hydroxylase mRNA was found only on GD 15.5, which led to the initial conclusion that the adrenal pathway of GC synthesis should be expressed only on GD 15.5 in the developing lung. Further studies showed that 3β-HSD1 and 21-hydroxylase were still expressed on GD17.5 [10,11]. This is important because the product of 21-hydroxylase, deoxycorticosterone (DOC), is known to bind the glucocorticoid receptor [12-15] with an IC_50_ value of 70 nM compared to 60 nM for corticosterone [15]. Therefore, the impact of 21-hydroxylase on lung development may be exerted during a longer period, but the exact developmental time remains to be characterized.

The aim of the present study was to deepen the knowledge on expression of “adrenal” glucocorticoid synthesis genes in the mouse lung during the perinatal period and to study expression of the 20α-HSD gene encoding for a steroid inactivating enzyme active on c21 steroids. An ontogenic study of P450scc, 3β-HSD1, 21-hydroxylase, and 11β-hydroxylase expression in the mouse developing lung was performed up to post-natal day (PN) 15. Genes encoding for 11β-HSD1 and 11β-HSD2 were included for comparison. In addition, the expression of *Akr1c18* gene, encoding for 20α-HSD, was included because 20α-HSD is active on the substrate (progesterone) and on the product (DOC) of 21-hydroxylase. This gene has never been studied in the lung. Because a sex difference in the timing of expression of the adrenal-like pathway of GC synthesis was proposed on GD 15.5 in the fetal lung [8], both sexes were studied separately. Finally, an ontogenic study of levels of 21-hydroxylase protein is also included.

## Results

### Expression of genes of the “adrenal” pathway of GC synthesis in the antenatal lung after GD 15.5 and in the post-natal developing lung

**Figure 2:**
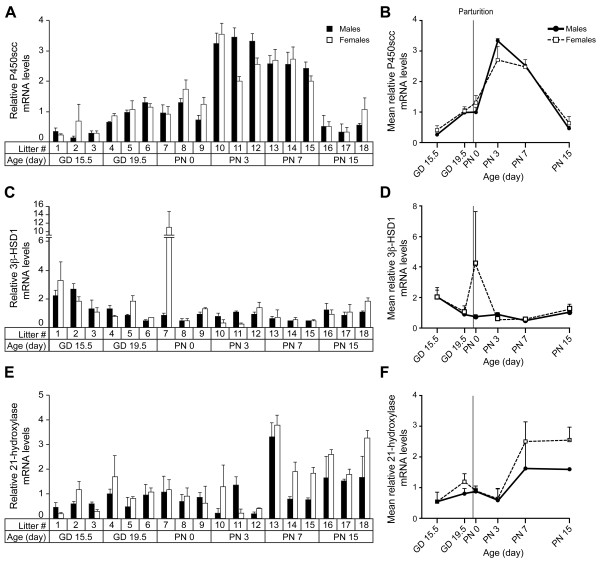
**Relative expression of P450scc, 3β-HSD1, and 21-hydroxylase genes according to sex and age in the mouse developing lung.** Relative expression values were determined by qPCR for P450scc **(A)**, 3β-HSD1 **(C)**, and 21-hydroxylase **(E)** in male and female pools of 3 litters for each age. The results are the mean of 2 PCR reactions (±SD). Then, the mean of expression (± SEM) for each sex and each age was calculated from the values presented in the histograms and the resulting curves are presented for P450scc **(B)**, 3β-HSD1 **(D)**, and 21-hydroxylase **(F)**.

The temporal and sexual expression profiles of P450scc, 3β-HSD1, 21-hydroxylase, and 11β-hydroxylase genes were determined using cDNA prepared from pooled male and female lung tissues (one pool/sex/litter) from GD 15.5 to PN 15. The 11β-hydroxylase transcript was detected at significant levels on GD 15.5, but not between GD 16.5 and PN 15 inclusively (data not shown). These data are in agreement with previously published data obtained between GD 15.5 and 18.5 [8,10], and on GD 17.5 [10]. Conversely, the P450scc, 3β-HSD1 and 21-hydroxylase genes were expressed after GD 15.5 at least up to PN 15 in the alveolar stage (Figure 2). In fact, mRNAs were detected for these three genes in all analyzed samples, exhibiting specific temporal expression patterns. A two-way ANOVA showed that P450scc mRNA levels varied significantly according to developmental age, but not sex (Table 1). In fact, a significant 18-fold increase was observed from GD 15.5 to GD 19.5, followed by a significant 2.3-fold increase between PN 0 and PN 3, and a marked significant 28-fold decrease from PN 7 to PN 15 (Figure 2A-B). Expression of 3β-HSD1 did not show significant temporal or sexual regulation, although a slight significant trend for decreased expression was observed over developmental time (Figure 2C-D and Table 1). A statistical analysis showed a significant influence of age but not of sex or sex*age on 21-hydroxylase expression (Table 1). Hence, 21-hydroxylase expression was stable from GD 15.5 to PN 3, presented a 4.3-fold increase between PN 3 and PN 7, and remained stable until PN 15 (Figure 2E-F). Inter-litter variability was somewhat higher on PN 7 and PN 15 for 21-hydroxylase, which might explain why no statistically significant sex difference was found despite the fact that all the 6 litters of this period showed higher expression levels for females compared to males (Figure 2E).

**Table 1:** Two-way analysis of variance (ANOVA) of expression of murine genes involved in C-21 steroid metabolism in the developing lung

**Gene**	**P values**		
	***Age***^**x**^ ***Sex***	***Age***	***Sex***
P450scc	0.6606	< 0.0001	0.3264
3β-HSD1	0.1387	0.0630	0.6889
21-hydroxylase	0.7968	0.0029	0.2728
20α-HSD	0.3141	0.0003	0.4667
11β-HSD1	0.3073	0.0104	0.8879
11β-HSD2	0.9989	< 0.0001	0.9233

### Sexual and temporal regulation of 20α-HSD expression in the antenatal and post-natal developing lungs

**Figure 3:**
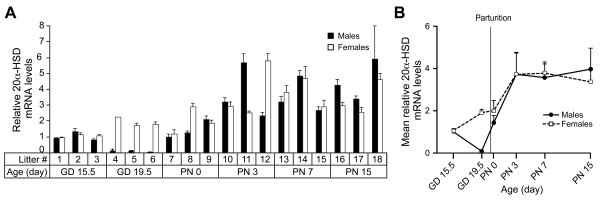
**Relative expression of 20α-HSD according to sex and age in the mouse developing lung.** Relative expression values were determined by qPCR for 20α-HSD **(A)** in male and female pools of 3 litters for each age. The results are the mean of 2 PCR reactions (±SD). Then, the mean of expression (± SEM) for each sex and each age was calculated from the values presented in the histograms **(B)**.

Progesterone and DOC inactivation can be achieved by 20α-HSD activity, which was observed in cytosolic fractions of adult mouse lungs [16] and in adult human lung slices [17]. However, an ontogenic study of 20α-HSD expression has never been reported in the developing lung. To perform this study, 20α-HSD mRNA levels were measured in mouse developing lung samples collected at various developmental times. Expression of 20α-HSD was observed in all samples analyzed from GD 15.5 to PN 15 (Figure 3). A statistically significant variation over developmental time was observed (Table 1) including a marked post-natal increase until PN 3 for both sexes. While no significant sex difference was detected by two-way ANOVA, a male-specific decrease of 19-fold before birth was observed (Figure 3). Expression levels of 20α-HSD were higher than those of 21-hydroxylase during near all the developmental period studied, with a difference of 5.4-fold in favor of 20α-HSD on PN 3.

### 11β-HSD1 and 11β-HSD2 expression profiles from GD 19.5 to PN 15

**Figure 4:**
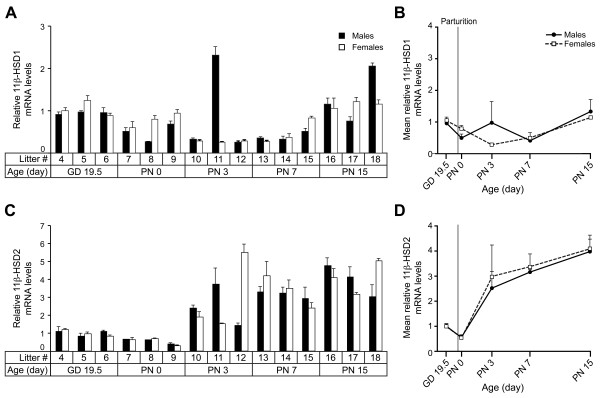
**Relative expression of 11β-HSD1 and 11β-HSD2 according to sex and time in the mouse developing lung.** Relative expression values were determined by qPCR for 11β-HSD1 **(A)** and 11β-HSD2 **(C)** in male and female pools of 3 litters for each age. The results are the mean of 2 PCR reactions (±SD). Then, the mean of expression (± SEM) for each sex and each age was calculated from the values presented in the histograms for 11β-HSD1 **(B)** and 11β-HSD2 **(D)**.

A qPCR study of 11β-HSD1 and 11β-HSD2 expression was reported in the fetal lung between GD 15.5 and 18.5 [8]. Here, the study covers the GD 19.5-PN 15 period. Only little changes were measured over developmental time for 11β-HSD1. In contrast, the 11β-HSD2 mRNA levels presented a marked statistically significant increase after birth (Figure 4 and Table 1).

### 21-Hydroxylase protein during lung development

**Figure 5:**
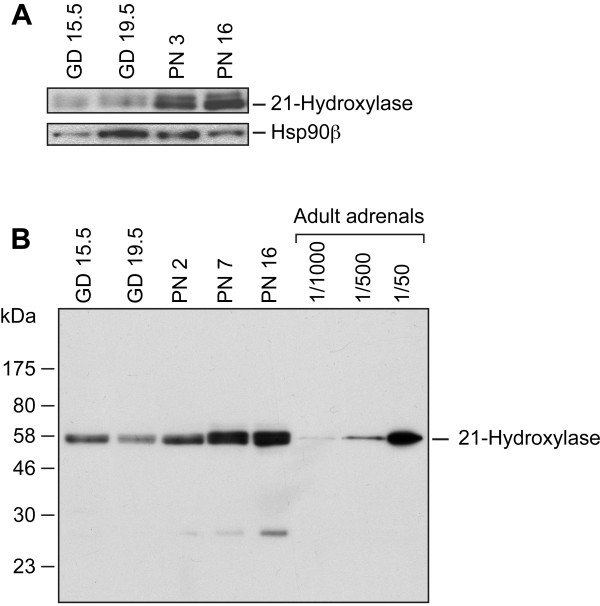
**21-hydroxylase protein in the mouse developing lung.** A Western blot analysis of the 21-hydroxylase protein is presented for whole lung protein extracts (50 μg) **(panel A)** and microsomal fractions (50 μg of microsomal proteins) **(panel B)** for the indicated developmental times. For the study of total protein extracts, levels of Hsp90β are shown as a loading control. Total protein extracts were prepared with the lungs from one individual for each time point, whereas microsomal fractions were prepared using a pool of lungs from all the fetuses/neonates of one litter for each time point. Microsomes from adult adrenals were used as control. The proportion of adrenal microsomal proteins loaded on the gel compared to the amount loaded for the lungs is indicated (1/1000 = 50 ng microsomal proteins, 1/500 = 100 ng, and 1/50 = 1 μg). Each pannel corresponds to a different gel.

The levels of the 21-hydroxylase protein were analyzed by Western blotting of whole lung protein samples and of microsomal fractions. Our data show that not only the 21-hydroxylase mRNA but also the 21-hydroxylase protein are present in the perinatal lung (Figure 5 and data not shown). Specific signals were higher in lung samples collected after birth compared to those obtained during gestation (Figure 5). Because 21-hydroxylase is a microsomal protein, microsome preparations were also studied. The upper unspecific signal observed with total protein extracts (Figure 5A) was undetected or close to the background with microsomal fractions (Figure 5B). The most intense signals were observed with samples from PN 7 and PN 16. This increase in 21-hydroxylase protein levels correlates with the increase in mRNA levels described above (Figure 2E-F). Intermediate protein levels were observed in samples from PN 3 (one individual) and PN 2 (pool from one litter). This can be explained by slight variations in mRNA levels from individual to individual or from litter to litter, as those observed in Figure 2E. No sex difference was observed at the specific developmental times tested, except on PN 15, when a slight difference in favor of females was observed (data not shown).

## Discussion

This is the first ontogenic study performed in the developing lung up to PN 15 studying expression of the four genes normally involved in the adrenal pathway of glucocorticoid synthesis from cholesterol. 11β-hydroxylase was previously studied in the developing lung after GD 15.5 (from GD 15.5 to GD 18.5 [8] and on GD 17.5 [10]) and no detectable levels of 11β-hydroxylase mRNA were found after GD 15.5. Here, studying the developing lung at several time points from GD 15.5 to PN 15, we conclude that 11β-hydroxylase mRNA can be found only on GD 15.5 in the developing lung. In contrast, P450scc, 3β-HSD1 and 21-hydroxylase mRNAs were found in all the analyzed samples from GD 15.5 to PN 15. Moreover, the 21-hydroxylase protein was observed in all the analyzed samples from GD 15.5 to PN 16. This is important because the substrate of this enzyme, progesterone, is present during gestation while the product of this enzyme, DOC, has the ability to bind the glucocorticoid receptor [12-15] with an IC_50_ value of 70 nM compared to 60 nM for corticosterone [15]. Therefore, the developing lung possesses the machinery to produce active glucocorticoids, at least during gestation, using circulating progesterone as a substrate.

A transient sex difference in 20α-HSD mRNA levels was observed on GD 19.5 in the developing lung, when the female values were 19-fold higher than the male values (Figure 3). A sex difference in 20α-HSD expression was also reported in the mouse adrenal [18]. In that tissue, 20α-HSD expression was restricted to the transient X-zone located between the cortex and the medulla. The presence of the 20α-HSD protein in adrenals was shown to correlate with the presence of the X-zone. On PN 10, no sex difference was observed in the lung in our experiments, whereas a sex difference was found in the adrenal where both 20α-HSD and the X-zone were absent specifically in the male tissue. Therefore, no relationship between the sex differences observed in the developing lung and in adrenals can be established. Sex differences in 20α-HSD mRNA levels with higher levels for females were also observed in the skin, liver, and kidney obtained from adult mice [19]. It was suggested that 20α-HSD may play a role in reducing intracellular levels of progesterone originating from the circulation [19]. In the lung, except on GD 19.5, no significant sex difference was observed in 20α-HSD mRNA levels, which is in line with the absence of sex difference in perinatal progesterone levels in rodents [20]. However, progesterone levels are decreasing in late gestation to reach very low levels after birth, which is not the case for 20α-HSD expression.

The expression of 20α-HSD in the developing lung is compatible with a function of this enzyme in modulation of progesterone receptor occupancy, but our data also suggest that another function should exist in the lung. Indeed, we observed a marked increase in 20α-HSD expression after birth in both sexes. Interestingly, this increase just preceded the increase in 21-hydroxylase expression. Therefore, we propose that 20α-HSD could play a role related to the glucocorticoid synthesis pathway. This enzyme can inactivate both the substrate (progesterone) and the product (DOC) of the 21-hydroxylase [18]. Thus, the lung 20α-HSD activity should influence glucocorticoid receptor occupancy.

**Figure 6:**
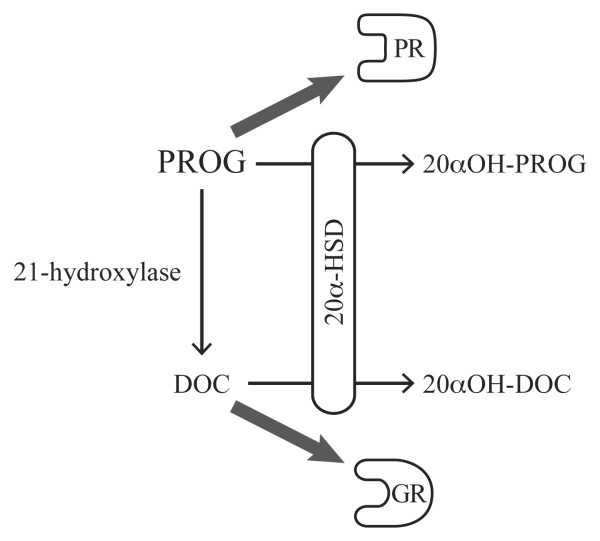
**Effects of 21-hydroxylase and 20α-HSD on progesterone receptor and glucocorticoid receptor occupancy.** PROG, progesterone; PR progesterone receptor; GR, glucocorticoid receptor.

The coexistence of GC synthesizing and inactivating activities in the developing lung strongly suggests regionalization of GC action. Intracrine as well as paracrine GC actions in the developing lung are compatible with our data. The concept of intracrine action was first demonstrated for dihydrotestosterone in the prostate [21,22]. It is defined as the action of newly-formed active steroids within the cells that synthesized them. As shown in Figure 6, the 21-hydroxylase activity changes the affinity of the substrate from progesterone receptor to glucocorticoid receptor, while the 20α-HSD activity controls the availability of active steroids (progesterone and DOC). Therefore, 20αHSD may be involved in the fine tuning of both progesterone receptor occupancy, through its impact on progesterone levels, and glucocorticoid receptor occupancy, through the regulation of the levels of the substrate (progesterone) and product (DOC) of 21-hydroxylase. This suggests an optimizing role for 20αHSD in lung development.

11β-HSD2 is expressed in aldosterone-selective target cells and prevents illicit occupation of mineralocorticoid receptor (MR) by GCs [23,24]. A role for mineralocorticoids is proposed in lung development [25] where co-expression of 11β-HSD2 and MR genes were found [26,27]. MR is proposed to be involved in the control of reabsorption of pulmonary fluid by ENaC, which is essential to the transition from uterine to aerial life at birth. However, ENaC is also modulated by glucocorticoids in the developing lung [28,29]. The increase in 11β-HSD2 mRNA observed in our experiment occurred after parturition and correlated well with the beginning of septation. Therefore, we propose that the process of septation may require a greater specificity of MR to mineralocorticoids.

## Conclusion

Our data show that expression in the developing lung of most of the genes involved in the adrenal-like glucocorticoid synthesis pathway is maintained beyond GD 15.5. The gene expression results are compatible with corticosterone formation on GD 15.5, and DOC formation after GD 15.5, both steroids having the capability to bind the glucocorticoid receptor. The presence of the 21-hydroxylase protein showed that the fetal lung contains the machinery catalyzing DOC formation from progesterone, which is present in the fetal circulation. Co-expression of 21-hydroxylase and 20α-HSD in the developing lung strongly suggests that this tissue does not act as a classical endocrine gland, but is rather a site of intracrine/paracrine actions.

## Methods

### Animals and tissue preparation

The protocol and procedures were approved by the animal care and use committee and the institutional review board of the Centre de Recherche du CHU de Québec (CPA-CRCHUQ; protocols no. 2005–156, 2008–071 and 2011–053). All animals were kept under a 12 h light/dark cycle and received water and feed ad libitum. Balb/c (*Mus musculus*) females at the estrus stage and males of the same strain were mated overnight (16 h). The day of copulatory plug was considered as GD 0.5 while the beginning of PN 0 corresponded to parturition. Pregnant females were kept into individual cages until sacrifice. All pregnant females were euthanized by CO_2_ inhalation. Pups were sacrificed either by decapitation following hypothermia-induced anesthesia (GD 15.5 to PN 5), or by intra-peritoneal injection of Euthanyl (PN 6 to PN 16; pentobarbital sodium, Bimeda-MTC, Cambridge, ON, Canada). Sex of fetuses/neonates was determined by gonadal morphology. Fetal and neonatal lungs were individually flash-frozen for use in qPCR and protein extraction. Adrenals from dams were also collected and flash-frozen.

### Real-time quantitative PCR of target and normalization genes

For each litter, flash-frozen tissues were pooled according to sex. Total RNA was extracted using Tri-reagent (Molecular Research Center, Cincinnati, OH, USA) according to the protocol of the manufacturer. Each RNA sample was purified on a CsCl gradient [30], using a TLA 120.2 rotor in an Optima MAX centrifuge (Beckman, Mississauga, ON, Canada). An aliquot of 4 μg of each total RNA sample was treated with DNase I (0.25 U/μg of RNA), and then reversed transcribed using Superscript II (Life Technologies, Burlington, ON, Canada) and hexameric random primers (pd [N]_6_, Life Technologies) for 50 min at 42°C in a final volume of 20 μl according to the protocol of the manufacturer. Reverse transcriptase was inactivated by heating at 70°C for 15 min. The resulting cDNAs were diluted tenfold before qPCR analyses. The same cDNA preparations were used for all of the analyzed genes. The qPCR reactions were performed on a LightCycler instrument (Roche, Montreal, QC, Canada) using LightCycler-FastStart DNA Master SYBR Green I kits (Roche). After qPCR enzyme activation (10 min, 95°C), PCR cycles were performed as follows: 5 Sec 95°C (denaturation); 5 sec at the annealing temperature; 20 Sec 72°C (elongation); 5 sec at the temperature of fluorescence intensity reading. The specific primer sequences and qPCR conditions are presented in Table 2 for: *Cyp11a1* (P450scc), *Hsd3b1* (3β-HSD1), *Cyp21a1* (21-hydroxylase), *Cyp11b1* (11β-hydroxylase), *Akr1c18* (20α-HSD), *Hsd11b1* (11β-HSD1), *Hsd11b2* (11β-HSD2), *Gapdh* (glyceraldehyde-3-phosphate dehydrogenase), and *Hmbs* (hydroxymethylbilane synthase, transcript variant 1). For each gene, the relative expression levels were calculated with a standard curve prepared using serial dilutions of an amplicon in 0.1X reverse transcription buffer. The use of this buffer for dilutions ensured comparable qPCR efficiencies between standard curve samples and lung cDNA samples, as reported [31]. Hmbs and Gapdh expression levels were obtained for all the samples and fed in the GeNorm program to generate a normalization factor for each sample [32]. For all the genes, the mean value obtained on GD 19.5 was fixed as onefold. The suitability of Hmbs and Gapdh as reference genes for normalization of developing lung samples had previously been confirmed [33].

**Table 2:** Analyzed murine genes and their specific real-time QPCR conditions

**Mouse genes**	**Accession number (NCBI)**	**Oligonucleotides (sense/antisense)**	**Amplicon length (bp)**	**QPCR conditions**^**a**^	
				**Ann. T (°C)**	**Acq. T (°C)**
*Cyp11a1*	NM_019779	ATCCGGGCTTCTTTCCCAATC/GGATGGGGTTCTCAGGCATC	249	64	86
*Hsd3b1*	NM_008293	TGCCAGGGCATCTCTGTTGTC/TCTGTTCCTCGTGGCCATTCA	220	64	84
*Cyp21a1*	NM_009995	TCACGACTGTGTCCAGGACTTG/TTCGTCTTTGCCATCCCTTTG	250	67	84
*Akr1C18*	NM_1340662.2	GCACCATAGGCAACCAGAAC/TCTCATTCATTTCCCAGTGTCTC	312	54	78
*Cyp11b1*	NM_001033229	CTGGGACAGTCCTCAATGTGA/ATCCGCACATCCTCTTTCTCTT	244	62	87
*Hsd11b1*	NM_008288	GGCCAGCAAAGGGATTGGAAG/TTTTCCCAGCCAAGGAGGAGA	401	66	85
*Hsd11b2*	NM_008289	TGGCTGACGTGGGACTGTCT/TTGGAGCAGCCAGGCTTGATA	277	63	87
*Gapdh*	NM_008084	GTCGGTGTGAACGGATTTG/AAGATGGTGATGGGCTTCC	215	61	84
*Hmbs*	NM_013551	GGAATGCATGTATGCTGTGGG/CAGGTACAGTTGCCCATCTT	208	59	85

^a^Ann. T, annealing temperature; Acq. T, acquisition (fluorescence intensity reading) temperature.

### Protein extraction

Protein extracts were prepared as previously described [33], with slight modifications. Briefly, lung tissues were lysed on ice using a micro-homogenizer (Power max AHS 200, VWR, Ville Mont-Royal, QC, Canada) and 50 to 300 μL of extraction buffer consisting of 50 mM Hepes, pH 7.5, 150 mM NaCl, 1 mM EDTA, 1 mM EGTA, 1 mM NaF, 1% (v/v) Triton X-100, 10% (v/v) glycerol, 20 mM β-glycerophosphate, 8 mM sodium pyrophosphate, and containing a protease inhibitor cocktail (Sigma–Aldrich, St. Louis, MO, USA; 1 mM AEBSF (4-(2-aminoethyl)benzenesulfonylfluoride), 0.8 μM aprotinin, 20 μM leupeptin, 40 μM bestatin, 15 μM pepstatin A, and 14 μM E-64, final concentrations). Lysates were centrifuged at 16 000 × g for 20 min at 4°C. Total protein concentrations in supernatants were determined using the Bio-Rad protein assay (Bio-Rad, Mississauga, ON, Canada) based on the method of Bradford. Samples were boiled 3 min in 0.06 M Tris–HCl, pH 6.8, 1% SDS, 3% β-mercaptoethanol, 10% glycerol, 0.025% bromophenol blue and then stored at −20°C until use.

For microsomes preparations, samples were homogenized by hand in an all-glass Dounce homogenizer fitted with a B-pestle in ice-cold buffer containing 20% (vol/vol) glycerol, 1.0 mM EDTA, and 4 mM potassium phosphate, pH 7.0 (KPBS). Homogenates were centrifuged at 12,000 × *g* for 20 min to remove cell debris. The resulting supernatants were centrifuged at 105,000 × *g* for 60 min. The pellets were resuspended in KPBS and centrifuged again at 105,000 × *g* for 60 min. The resulting pellets were resuspended in the Triton X-100-containing extraction buffer described above, and saved as microsomes-enriched extracts. Protein concentrations in microsomal fractions were determined as described above.

### Western blot analysis and specific antibodies

Lung protein extracts were separated by sodium dodecyl sulfate-polyacrylamide gel electrophoresis (SDS-PAGE) using 10% (wt/vol) acrylamide. Then, proteins were transferred to a nitrocellulose membrane (Bio-RAD). Membranes were blocked 2 h at room temperature with 5% (w/v) fat-free milk powder in PBS (1× PBS: 137 mM NaCl, 2.68 mM KCl, 4.30 mM Na_2_HPO_4_, 1.47 mM KH_2_PO_4_) containing 0.1% (v/v) Tween 20. Membranes were then incubated 1 h at room temperature with either a mouse anti-rat 21-hydroxylase monoclonal antibody (1:3000; Chemicon International Inc., ON, Canada) or a rabbit anti-Grp78 (Hsp90β) polyclonal antibody (1:100; Santa Cruz Biotechnology, Santa Cruz, CA, USA) in 1X PBS/Tween 20 containing 2.5% (w/v) fat-free milk. After washing in 1X PBS/Tween 20, bound antibodies were visualized with horseradish peroxidase-conjugated goat anti-mouse or goat anti-rabbit antibodies (1:5000; Jackson ImmunoResearch Laboratories, West Grove, PA, USA) using a Lumi-Light Western Blotting Substrate (Roche Applied Science). Then, antibodies were removed by washing in 62.5 mM Tris–HCl, pH 6.8, 2% (w/v) SDS, 0.7% (v/v) β-mercaptoethanol for 30 min at 60°C. The stripping efficiency was validated with a Lumi-Light Western Blotting Substrate. Membranes were first used to detect 21-hydroxylase, stripped, and then re-probed for Hsp90β.

### Statistics

The effects of age and sex on target gene expression data was assessed by a randomized-block 2-way ANOVA, male and female pools of the same litter being matched. The age of the studied litters was considered as a fixed factor. To ensure that assumptions of normality and homogeneous variances were respected, the dependent variable (expression) was either untransformed (P450scc, 20α-HSD), transformed into its natural log (21-hydroxylase, 11β-HSD1 and 11β-HSD2) or transformed into its reciprocal (3β-HSD1). For each gene, comparisons between the mean expression values (calculated with data from both sexes) at specific age-points were performed using the Bonferroni test on selected pairs. The differences were considered statistically significant when p < 0.05, whereas for 0.1 > p > 0.05, the differences were considered to present a significant trend.

## Abbreviations

GD: Gestation day; PN: Post-natal day; HSD: Hydroxysteroid dehydrogenase; GC: Glucocorticoid; DOC: Deoxycorticosterone; KO: Knockout; SP: Surfactant protein; MR: Mineralocorticoid receptor.

## Competing interests

The authors declare that they have no competing interests.

## Authors’ contributions

EB carried out the sample collection, performed the qPCR and Western blot experiments, performed the statistical analyses, and participated in the design of the study and in the drafting of the manuscript. PP participated in the design of the study and drafted the manuscript. YT conceived the study, participated in its design, and helped to draft the manuscript. All authors read and approved the final manuscript.
